# Exosomes: A potential tool for immunotherapy of ovarian cancer

**DOI:** 10.3389/fimmu.2022.1089410

**Published:** 2023-01-18

**Authors:** Xiangjin Gong, Hao Chi, Dorothee Franziska Strohmer, Alexander Tobias Teichmann, Zhijia Xia, Qin Wang

**Affiliations:** ^1^ Southwest Medical University, Luzhou, China; ^2^ Clinical Medical College, Southwest Medical University, Luzhou, China; ^3^ Department of General, Visceral, and Transplant Surgery, Ludwig-Maximilians-University Munich, Munich, Germany; ^4^ Sichuan Provincial Center for Gynecology and Breast Diseases (Gynecology), Affiliated Hospital of Southwest Medical University, Luzhou, China

**Keywords:** exosome, ovarian cancer, immunotherapy, tumor microenvironment, biomarker

## Abstract

Ovarian cancer is a malignant tumor of the female reproductive system, with a very poor prognosis and high mortality rates. Chemotherapy and radiotherapy are the most common treatments for ovarian cancer, with unsatisfactory results. Exosomes are a subpopulation of extracellular vesicles, which have a diameter of approximately 30–100 nm and are secreted by many different types of cells in various body fluids. Exosomes are highly stable and are effective carriers of immunotherapeutic drugs. Recent studies have shown that exosomes are involved in various cellular responses in the tumor microenvironment, influencing the development and therapeutic efficacy of ovarian cancer, and exhibiting dual roles in inhibiting and promoting tumor development. Exosomes also contain a variety of genes related to ovarian cancer immunotherapy that could be potential biomarkers for ovarian cancer diagnosis and prognosis. Undoubtedly, exosomes have great therapeutic potential in the field of ovarian cancer immunotherapy. However, translation of this idea to the clinic has not occurred. Therefore, it is important to understand how exosomes could be used in ovarian cancer immunotherapy to regulate tumor progression. In this review, we summarize the biomarkers of exosomes in different body fluids related to immunotherapy in ovarian cancer and the potential mechanisms by which exosomes influence immunotherapeutic response. We also discuss the prospects for clinical application of exosome-based immunotherapy in ovarian cancer.

## 1 Background

Ovarian cancer is one of the three major gynecological malignancies, accounting for approximately 2.5% of all female cancers (1). The 5-year survival rate for early-stage I ovarian cancer is 70%, compared to less than 29% for advanced stage III or IV (1). Currently available treatments for ovarian cancer mainly include chemotherapy, radiotherapy, surgery, and targeted therapy (2). Among them, chemotherapy and radiotherapy are the most effective means to treat ovarian cancer in clinical practice; however, they have disadvantages including adverse reactions, drug resistance, and long-term complications (3). In the context of significant advances in drug screening technology (4), there has been increasing interest in the development of oncology drugs that harness new cancer treatment strategies to overcome these problems. Cancer immunotherapy is a therapeutic method to control and eliminate tumors by regulating the immune function of tumor cells (5). Cancer immunotherapy can enhance the immune system and facilitate a durable response, which is suitable for a variety of cancers and can harness the immune system to reactivate the anticancer immune response that overcomes tumor escape (6). Treatments include adoptive cell transfer, nonspecific immune stimulation, vaccination strategies, and immune checkpoint blockade (2).

In recent years, exosome-based immunotherapy for ovarian cancer has become a research hotspot. Exosomes refer to small membrane vesicles with a diameter of 30–100 nm, which contain complex RNA, proteins, lipids, sugars, and nucleic acids (7, 8). Exosomes act on receptors on the cell membrane or directly fuse with the membrane of target cells to participate in local and distant information conduction (9). Exosomes can also be used as potential biomarkers for ovarian cancer (10). Meanwhile, exosomal miRNAs are biomarkers for the diagnosis and prognosis of ovarian cancer (11). Indeed, increased cytoplasmic expression of CD24 is a marker of reduced survival in patients with serous adenocarcinoma of ovarian cancer and is one of the biomarkers of epithelial ovarian cancer (12). In addition, claudin-4 protein is released by ovarian cancer cells and is highly expressed in the peripheral circulation of of ovarian cancer patients. Therefore, exosomes are valuable as screening biomarkers for the detection, diagnosis, and prognosis of ovarian cancer (13).

**Figure 1:**
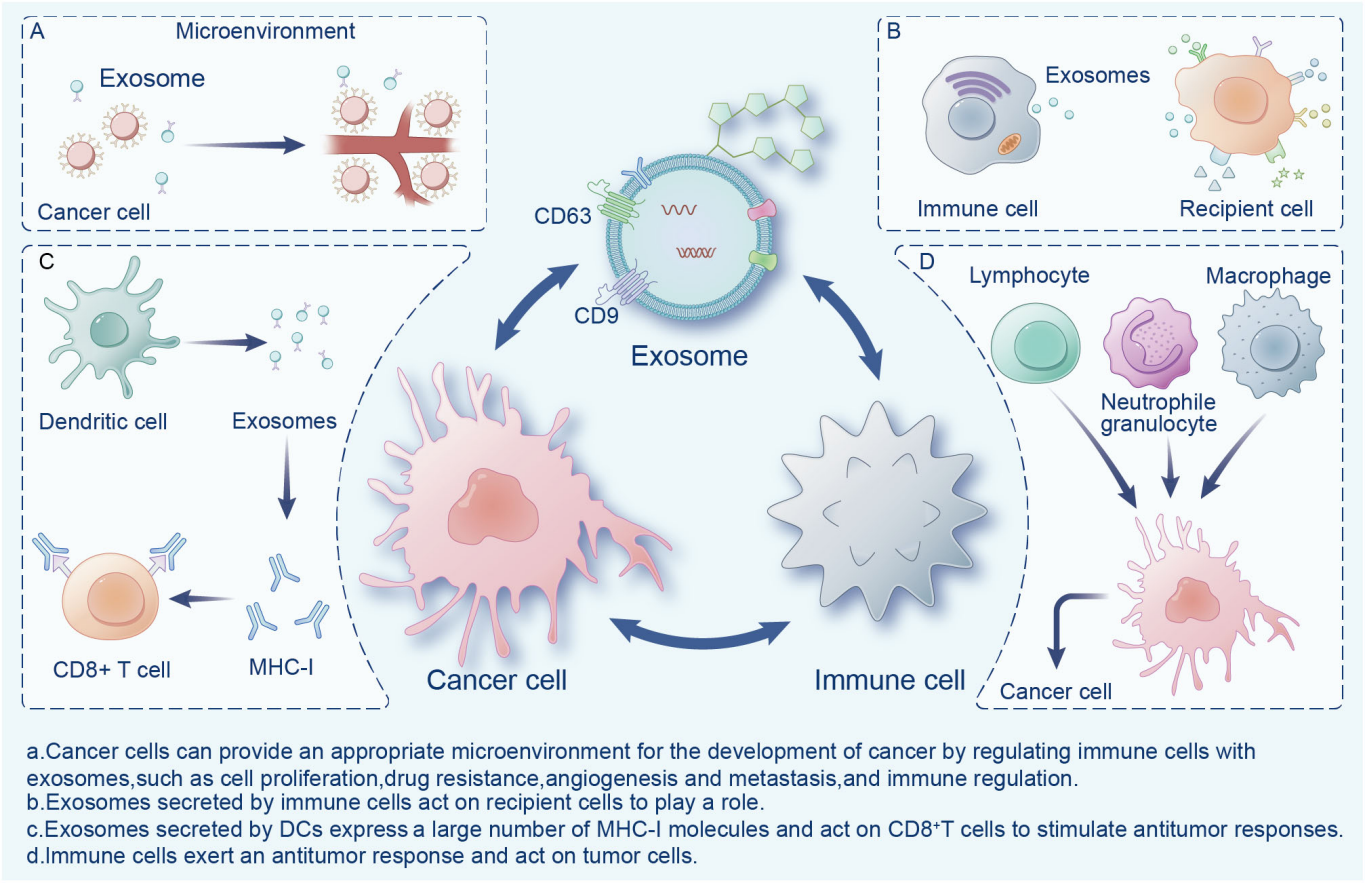
Interactions between exosomes, cancer cells, and immune cells in the tumor microenvironment.

Exosomes are widely present in the tumor microenvironment (TME), which consists of surrounding non-malignant cells, non-cellular components, extracellular matrix (ECM), and signaling molecules (14). Exosomes are a double-edged sword in the TME, playing an important role in the mutual regulation of tumor and immune cells (**Figure 1**). Cancer cells can provide an appropriate microenvironment for the development of cancer by regulating immune cells with exosomes, such as *via* cell proliferation, drug resistance, angiogenesis and metastasis, and immune regulation (15). Meanwhile, exosomes secreted by cancer cells can change different types of stromal cells, and promote the growth and invasion of cancer cells, as well as tumor angiogenesis (16). In contrast, immune cells activate immune responses in the TME through exosomes (17). Exosomes exhibit immunogenicity and cell transfer function (18). Exosomes show high antitumor activity in a variety of tumors, promote the expansion of regulatory T cells, inhibit the proliferation and activation of CD8+ T cells, and play an immunosuppressive role. Researchers have found that dendritic cells (DCs) and tumor-secreted exosomes enable antigen presentation and T cell stimulation by expressing numerous major histocompatibility complex class I molecules (MHC-I) and tumor markers, and trigger CD8+ T cell-dependent antitumor responses *in vitro* and *in vivo* (19). Therefore, exosomes have great potential in cancer immunotherapy and may become the most effective vaccine to stimulate the anti-cancer immune response and serve as a vehicle for targeted anti-gene drugs (20, 21).

This review focuses on exosomes as biomarkers in tumor diagnosis, their role in the TME, and as immunotherapy tools for ovarian cancer.

## 2 Exosomes in tumor diagnosis

**Figure 2:**
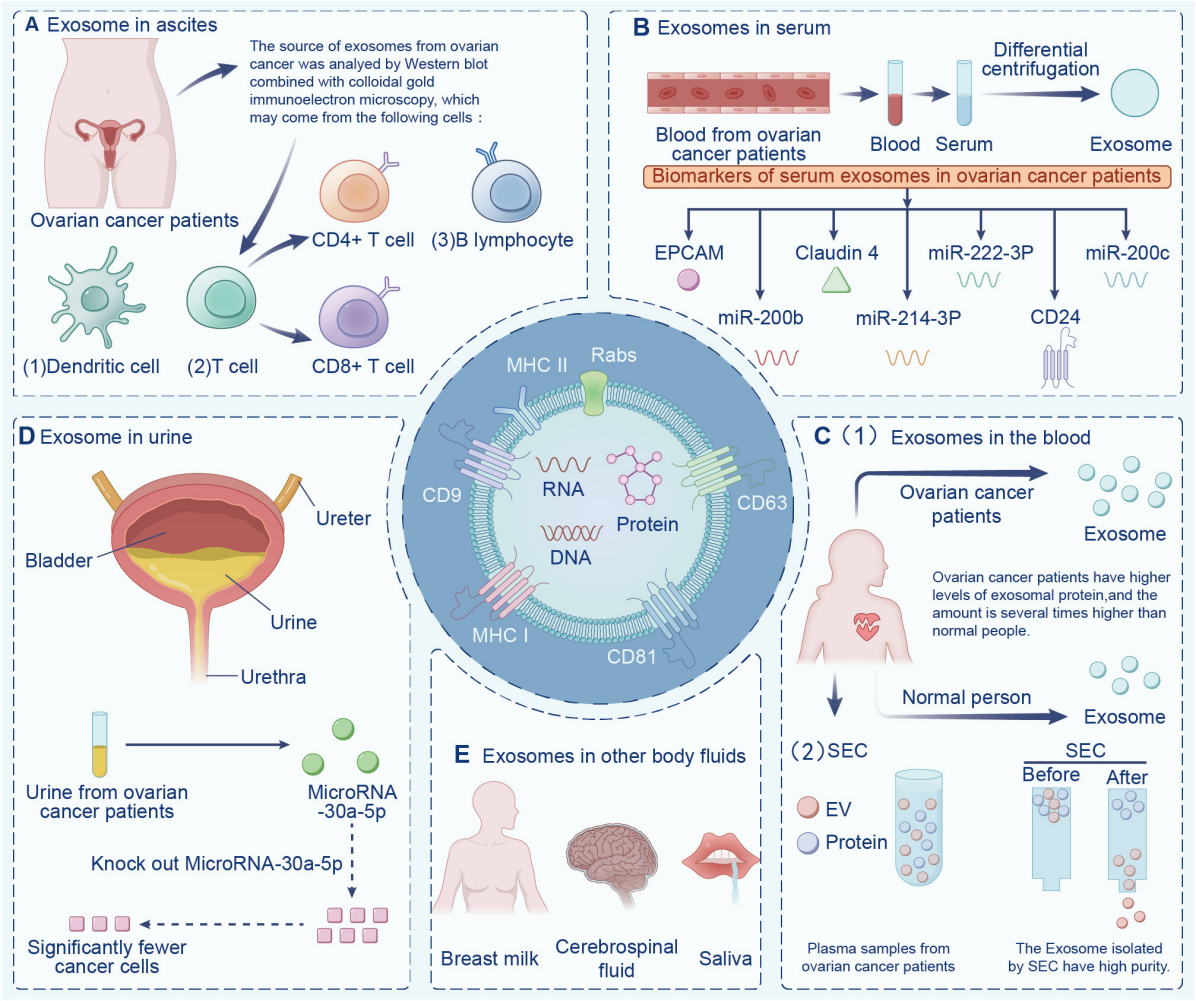
Exosomes in different body fluids. Exosomes are widely found in a variety of body fluids, including **(A)** ascites, **(B)** serum, **(C)** blood, **(D)** urine, and **(E)** in other fluids have the potential to be immune markers due to their particularity.

Exosomes are widely found in various body fluids (including ascites, blood, urine, emulsion). In the last decade, exosomes have been suggested to have potential as immunotherapy markers due to their particularity (22, 23). First, exosomes may be superior to some traditional diagnostic methods in terms of sensitivity and specificity, and exosomes contain a variety of bioactive molecules, resulting in less interference (24). Second, exosomes are highly stable and do not degrade in the extracellular environment. Finally, exosomes are widely present in various body fluids. Indeed, the serum exosomes of patients with ovarian cancer contain significantly more circ-0001068 (a novel biological marker) than those of healthy volunteers (25). Studies have found that exosomes in ascites are related to tumor invasion, metastasis, and survival time, and exosomes are highly expressed in ascites (26). Additionally, exosomes from ovarian cancer ascites containing CD147 could be used to monitor treatment response (27). Currently, exosome-based diagnostic kits for clinical diagnosis have been approved by the US Food and Drug Administration (28). This section summarizes exosomes in different body fluids (**Figure 2**).

### 2.1 Exosomes in ascites

Various factors contribute to the composition of cancer ascites, including tumor cells, fibroblasts, immune cells, and non-cellular items, such as cytokines, proteins, and exosomes (29), which together regulate the malignant phenotype and biological behavior of tumor cells (30). Numerous tumor-derived exosomes accumulate in the ascites of cancer patients, and ascites-derived exosomes present as novel substances of cancer-rejecting immune antigens, which opens up a new direction in the field of cancer immunotherapy (31). The complexity of ascites determines the multi-origin of ovarian cancer exosomes. Indeed, the source cells of ovarian cancer exosomes may include T cells, B cells, DCs, and ovarian cancer cells (32). Meanwhile, exosomes play crucial roles in tumor immune escape by inducing apoptosis of immune cells (33, 34). Other studies have found that L1 cell adhesive molecules can effectively inhibit the spread of ovarian cancer cells, and CD24 protein is a biomarker of poor prognosis of ovarian cancer (35, 36). Additionally, exosomes has been shown to significantly promote the migration of ovarian cancer cells and increase chemotherapy resistance under a hypoxic environment (37). In this way, exosomes can serve as potential biomarkers of ovarian cancer cells’ proliferation, metastasis, and immune escape (38).

Peng et al. isolated exosomes from ascites of patients with ovarian cancer to stimulate PBMCs, and tested the cytotoxicity of PBMCs on ovarian cancer cells (39). Even though exosomes themselves did not affect the invasion and metastasis of ovarian cancer cells, they could impair the cytotoxicity of PBMC in the presence of DCs, thereby achieving anti-tumor immunity. Secretions isolated from patients with ovarian cancer without chemotherapy or radiotherapy using ultracentrifugation and the fluid secretion of body surface markers were analyzed. The results showed significant levels of CD63 and CD9 expression on the surfaces of exosomes in ovarian cancer ascites. Furthermore, the researchers demonstrated that ascites exosomes affect the invasion and metastasis of ovarian cancer cells. Exosomes from patient ascites transferred miR-6780b-5p to ovarian cancer, thereby promoting the metastasis, invasion, and proliferation of ovarian cancer cells (26). Two cargo proteins, CD24 and EpCAM, were found in exosomes from malignant ascites of patients with ovarian cancer. Studies have demonstrated that CD24 is a diagnostic biomarker for poor prognosis of ovarian and other cancers (40). Therefore, exosomes possess potential value in the diagnosis, metastasis, and progression of patients with ovarian cancer.

### 2.2 Exosomes in serum

New advances have been made in the early diagnosis of ovarian cancer. Recent studies have illustrated that exosomes derived from ovarian cancer contain miRNA, EpCAM, CD24, and other molecules (41–43). Several cancers are characterized by overexpression of EpCAM, which is associated with the proliferation of epithelial cells during tumorigenesis and development (44–46). Glycosylphosphatidylinositol (GPI) links CD24 to the cell surface, and is present in multi-vesicular bodies in the cytoplasm. The quantity of CD24 is closely correlated with the amount of EpCAM in the cytoplasm (47, 48). Exosomes release CD24 into the extracellular microenvironment, which can serve as a tumor marker and predict prognosis for ovarian cancer (49). Increased expression of CD24 indicates an increased invasion rate, poor prognosis, and reduced survival rate of patients with ovarian cancer (42). EpCAM has been detected in exosomes isolated from serum of patients with ovarian cancer, which confirmed the presence of diagnostic miRNAs in exosomes (50). A further study showed that serum exosomes from patients with ovarian cancer contained higher levels of mRNA and miRNA than those from healthy people (51), which provided a basis for tumor-derived exosomes to participate in the transport of genetic material between cells. This also demonstrates that diagnostic miRNAs in serum exosomes of patients with ovarian cancer can be used for the diagnosis of ovarian cancer (52, 53).

A study by Shen et al. found a positive correlation between tumor stage and claudin-4 expression in serum exosomes from patients with ovarian cancer (54). In ovarian cancer tissues with a high level of malignancy, Yang et al. extracted exosomes from serum and found miR-214-3p was highly expressed, which might serve as a biomarker for ovarian cancer diagnosis and prognosis in serum exosomes (55, 56). In patients with ovarian cancer, serum exosome miR-222-3p was more strongly expressed than in healthy women (57, 58). Patients with intermediate and advanced ovarian cancers had higher levels of exosomal miR-200b and miR-200c expression than those with early-stage ovarian cancers (59, 60). It is common for patients with ovarian cancer to develop malignant ascites as their disease progresses; thus, non-invasive detection based on serum exosome miRNA profile has potential value as a new biomarker for early screening and diagnosis of ovarian cancer. Ovarian cancer serum contains significantly more exosomes than benign ovarian tumor serum and normal serum (61). Additionally, patients with advanced ovarian cancer have been found to have significantly more proteins in their exosomes than those with early ovarian cancer (62, 63). It is reasonable to assume that exosome protein contents can be used as a biomarker to identify ovarian cancer stages.

### 2.3 Exosomes in plasma

Different proteins and Rnas have different effects on immunotherapy. Under normal physiological conditions, programmed cell death protein 1 (PD-1) prevents autoimmunity and keeps T-cell responses within the required physiological range to prevent excessive inflammatory responses from harming the body. But in cancer, PD-1 protects tumor cells from anti-tumor T cell responses, leading to tumor immune escape (64). Cytotoxic T lymphocyte-associated protein 4 can act as an immune checkpoint and down-regulate immune response. It is currently considered as a promising immunosuppressive drug. MiRNA-424 in extracellular vesicles of tumors inhibits CD28-CD80/86 co-stimulatory pathways in T cells and dendritic cells, leading to resistance to immune checkpoint blocking. Modified extracellular vesicles that knock down this miRNA can enhance the efficacy of cancer immune checkpoint suppression therapy (65). The composition of plasma is complex and contains various proteins and RNA which may affect tumor immune response. Serum is a fluid collected after blood clotting, which screens out fibrinogen and clotting factors, and increases clotting products. In the process of coagulation, platelets secrete a large number of exosomes, which affects the accuracy of research results (66).

The overall protein level of exosomes in the plasma of patients with ovarian cancer is higher than that of benign tumors or healthy people, and the expression of miRNA in the exosomes in cancer cell lines, tumor tissues, and plasma has been shown to be significantly different (67). The plasma samples contain abundant soluble proteins (such as albumin and fibrinogen) as well as lipoprotein particles and exosomes. Circulating immunoglobulins in plasma bind to tumor-derived exosomes, inducing antibody responses to tumor antigens and weakening complement-mediated cytotoxicity against tumor cells (68). Plasma exosome PD-L1 enables cancer cells to evade antitumor immunity. Exosomes deliver PD-L1 from the original cancer cells to other cell types with low or no expression of PD-L1, inhibiting systemic antitumor immunity (64). In addition, specific circulating mirnas (such as miR-21-5p, miR-24-3p, etc.) in the whole plasma and plasma exosomes can be used as predictive biomarkers of anti-PD-1/PD-L1 therapeutic response (69). Plasma lncRNA HOTAIR has been shown to promote the development of tumor and influence the poor prognosis of tumor (70). Due to the complex composition of plasma samples, the influence of free proteins on exosome separation cannot be ignored (71). Researchers found that when compared to conventional biomarkers, exosomes can be considered to have far greater stability (72), as well as being available at considerably higher volumes in the plasma of patients with ovarian cancer compared to healthy people (73, 74).

If the exosomes isolated from plasma contain a large number of free heterotrimeric proteins, subsequent proteomic data analysis will be seriously affected. Not only is the number of detected exosomal proteins limited, but the reduced number of detected proteins leads to a decrease in the abundance of most of the major proteins, which affects the subsequent differential analysis and validation. At present, how to best isolate exosomes is a great challenge. Among the existing exosome separation technologies (75), most researchers prefer differential centrifugation. However, the number of proteins detected after the separation of plasma exosomes by differential centrifugation is less than 300, and the heterotrimeric proteins cannot be effectively removed. Another exosome separation technique, molecular size-based exclusion chromatography (SEC), can obtain exosomes with high purity, which is sufficient for subsequent nucleic acid studies (76, 77). Therefore, the SEC method is being increasingly favored by exosome researchers. However, the SEC method can lead to lipoprotein impurity contamination and has room for improvement.

### 2.4 Exosomes in urine

The study found that ovarian cancer has unique metabolic characteristics in urine, so urine can be used as the basis for clinical diagnosis and classification of ovarian cancer (78). MiR-15a was significantly up-regulated and let-7a was down-regulated in the urine of ovarian cancer patients, showing potential as a specific diagnostic marker for ovarian cancer (79). The sensitivity and specificity of HMGA1 in ovarian cancer urine are high, and the detection of HMGA1 level in urine can be used as the basis for the diagnosis of serous ovarian cancer (80). Serum biomarker CA125 is an FDA-approved biomarker for ovarian cancer, and urine HE4 is the first marker after CA125 to be approved by the FDA for the diagnosis of ovarian cancer (81). Urinary mesothelin is also a good diagnostic marker for ovarian cancer (82). However, the negative news is that mucinous ovarian cancer does not express HE4, but CA125. In other words, these markers are limited and can only be used as diagnostic markers for specific types of ovarian cancer. In addition to these substances, all types of ovarian cancer urine contains rich and easily enriched exosomes with stable structure. Urinary exosomes are small vesicles secreted into the urine by renal epithelial cells (83) *via* two mechanisms: one is the direct shedding or budding of cell membrane, and the other is the fusion of intracellular multivesicular bodies with the plasma membrane, in which the specific exocytotic vesicles secreted by plasma membrane are urine exosomes. The separation methods of urine exosomes include simple high-speed centrifugation (84), sucrose gradient high-speed centrifugation, and reagent precipitation (85). Isolated urine exosomes have been found to have signature proteins and corresponding particle sizes by immunoelectron microscopy and nanotracer analysis.

Proteins of urine exosomes are derived from glomeruli, renal tubules, prostate, and bladder cells, indicating that exosomes in urine are secreted by cells of the kidney and other urinary organs (86, 87). In addition to proteins, urine exosomes also contain nucleic acids. Indeed, it has been found that urine exosomal RNA is more advantageous than total urine mRNA as a marker of kidney disease (88). Because the membrane structure of urine exosomes can reduce the degradation of RNA enzymes, their stability is higher (89). Additionally, RNA quality analysis and high-throughput sequencing of urinary exosomes revealed that the most important RNA in urinary exosomes is small RNA (90), including miRNA, which is a small non-coding RNA that plays a regulatory role in mRNA processing. RNA, especially miRNA, not only has important applications in the field of renal biomarkers (91), but also suggests the value of exosomes as a basis for biological therapy. MiR-92a is significantly up-regulated in the urine of patients with ovarian cancer, and can be used as a diagnostic marker for ovarian cancer (15). In addition, urinary exosome miR-106b was significantly down-regulated in ovarian cancer samples, showing certain diagnostic potential (92). Urinary exosome miRNA-21 has been widely studied as an emerging biomarker for the diagnosis of prostate cancer, which induces cancer cell proliferation and invasion by regulating the expression of multiple tumor related genes (93). Urinary exosome miR-4516 also marks premature ovarian failure (94).

Exosomes promote the development of ovarian cancer by regulating the biological behavior of tumor cells. Zhou et al. found that microRNA-30a-5p was highly expressed in urine exosomes of patients with ovarian cancer, and once the miR-30a-5p gene was knocked down, the proliferation and migration of ovarian cancer cells could be significantly inhibited (56, 95).

## 3 Roles of exosomes in the TME

**Figure 3:**
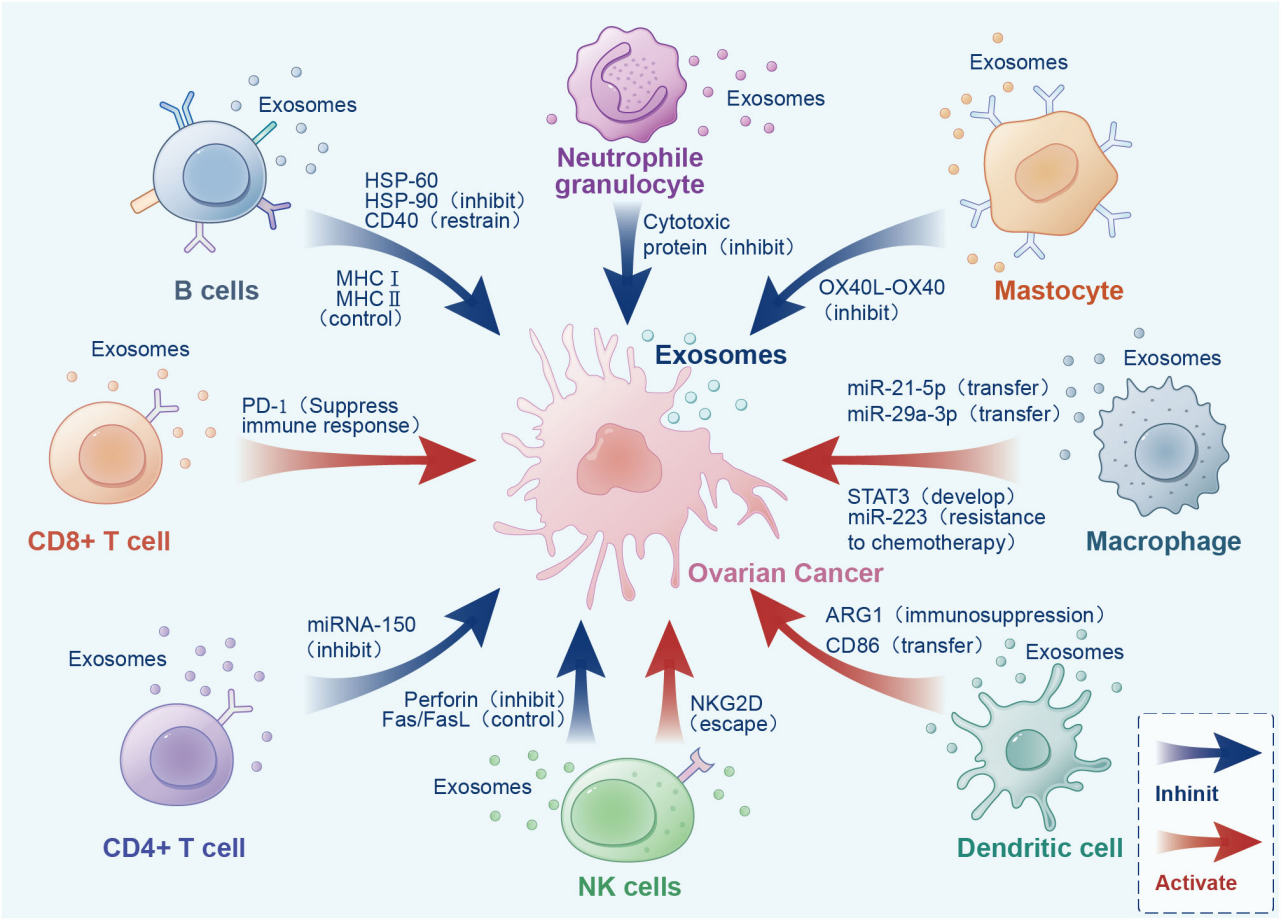
Bidirectional effects of exosomes from different sources in the tumor microenvironment.

Increasing experiments have proved that cancer cells secrete more exosomes than normal cells. Different exosomes carry different proteins, miRNAs, and other substances (96). Exosomes in ovarian cancer play an important role in tumor occurrence and development (97). We will describe the role of exosomes secreted by different cells in the TME (**Figure 3**, **Table 1**).

**Table 1:** Role of exosomes from different cell sources in the tumor microenvironment.

Source of exosomes	Material	Object	Role	Reference
CD8+T cells	mRNA, miRNA	Malignant tumor	Affects tumor development	(98)
	miR-765/PLP2	UCEC	Inhibition of estrogen to promote UCEC	(99)
CD4+T cells	FasL	T cells	Induced T cell apoptosis and immune disorders	(100)
	miRNA-150	Lymphoid tissue	Serum markers	(101)
B cells	DC vaccine	T cells	Inhibition of immune suppressive cytokine production	(102)
NK cells	Perforin Fas/FasL	Tumor cells	Cytotoxicity, tumor cell apoptosis	(103)
	Immune components	Tumor cells	Immunomodulatory, reverse tumor immune suppression	(104)
DCs	MHC I, MHCII	TME	The immune response is dysfunctional	(105)
Neutrophil granulocyte	Cytotoxic protein	Tumor cells	Induction of apoptosis	(106)
	Adriamycin	Glioma	No limitations, promising treatments	(107)
Macrophages	miRNA	Ovarian cancer	Immune suppression, promote cancer development	(108)
	miR-193a-5p	Tumor cells	Promote tumor invasion	(109)
	IncRNA	Cancer cells	Aerobic glycolysis, anti-apoptosis ability of cells	(110)
Mast cells	KIF protein	Tumor	Promote cancer cell proliferation	(111)

UCEC, Endometrial cancer.

### 3.1 Exosomes released by tumors

Studies have found that exosomes carrying relevant molecules (including proteins and miRNAs) can be released from tumor cells and stromal cells in the TME, and interact with immune cells in TME to conduct information transmission (112). Exosomes released by tumors provide a suitable microenvironment for tumor cells, but also inhibit the metastasis, occurrence, and development of tumor cells (38, 113).

Ovarian cancer, unlike other cancers, invades the abdominal cavity through ascites (114). Early ascites contains isolated tumor cells, various immune cells, mesothelial cells, and tumor-associated exosomes. These exosomes carrying protein signals specific for ovarian cancer can be isolated from the ascites and serum of patients with ovarian cancer (1). These exosomes can be used as biomarkers for early diagnosis of ovarian cancer. Exosomes secreted by ovarian cancer not only reveal the role of early malignant tumors, but also promote metastasis (115).

A common prerequisite for ovarian cancer metastasis is the formation of a premetastatic niche, which is the microenvironment formed at the distal sites by factors including exosomes secreted by ovarian cancer (116). The premetastatic niche requires immune suppression and evasion, angiogenesis, cancer-associated fibrocytes and tumor macrophages that reshape the stroma of the primary site (117). Studies have also shown that exosomal miRNAs play an indicative role in the pre-transfer niche (118). Exosomes translocate exfoliated miRNAs to tumor cells and their associated macrophages (TAM) and mesothelial cells to regulate the gene expression of target genes (119). However, ovarian cancer metastasis still faces an important barrier to the immune system. The mechanism of anti-tumor immune response in patients with ovarian cancer is disrupted precisely because exosomes suppress the immune response against the tumor (120). Exosomes secreted by ovarian cancer can induce T-cell arrest to achieve immune escape from cancer cells (121). Cytokines are closely associated with tumor progression and immune response (122, 123), and there is evidence that IL-6 promotes distant metastasis in ovarian cancer (124). In other cancers, exosomes have been found to induce IL-6 production in monocytes through a Toll-like receptor (TLR). IL-6 is then involved in signaling and transcription of activator 3 (STAT3) in immune cells, stromal cells, and tumor cells to complete immune escape and realize cancer cell metastasis (125). Additionally, exosomes evade immune surveillance by inhibiting NK cell function (126), inhibiting dendritic cells differentiation (127), and promoting myeloid inhibitory cell differentiation (128). In the omental TME, exosomes secreted by stromal cells contain miR-21, which can change the malignant phenotype (cancer cell movement and invasion) of metastatic ovarian cancer cells, indicating a new direction for the inhibition of ovarian cancer metastasis (129).

At the same time, exosomes from ovarian cancer can induce apoptosis of DCs, hematopoietic stem cells, and peripheral blood lymphocytes in the microenvironment, and inhibit anti-tumor immune response (130). In a study on exosomes from ovarian cancer researchers prepared two sets of culture groups, one with exosomes from malignant ovarian cancer ascites and the other with peritoneal lotions from benign ovarian cancer patients. Normal peripheral blood lymphocytes were added for co-culture and then lymphocytes were extracted for low gene expression analysis. The results showed that 26 immunosuppressive genes were overexpressed in lymphocytes of the malignant ovarian cancer ascites culture group compared to the benign ovarian cancer group, indicating that exosomes inhibit the immunity of lymphocytes through direct interaction with leukocytes (1). Exosomes have also been shown to silence immune cells in the TME, while their phosphatidylserine has been shown to inhibit T cell activation and shorten the growth phase of ovarian cancer (131).

### 3.2 Exosomes derived from T cells

Different T cells have different cell surface differentiation antigens (CD), which can be divided into CD4+ and CD8+ subsets. CD4+ T cells recognize exogenous antigenic peptides presented by MHC class II molecules, while CD8+ T cells recognize endogenous antigenic peptides presented by MHC class I molecules (132). The number and ratio of T lymphocyte subsets can be used as important indices of cellular immune function (133) in the context of viral infection, cancer, autoimmune diseases, and organ transplant, playing an important role in guiding treatment and prognosis (134). Such as malignant tumors, hereditary immunodeficiency diseases, AIDS, and CD4+T lymphocyte depletion in patients on immunosuppressive drugs (135). An increase in CD8+T cells may indicate autoimmune disease or chronic viral infection, such as chronic active hepatitis or tumor (136). Additionally, if the ratio of CD4/CD8 after transplantation is increased compared to that before transplantation, the patient may have suffered a rejection reaction (137). In the field of tumor immunotherapy, exosomes derived from T lymphocyte subsets have attracted extensive attention based on the various indicative effects of CD8+ and CD4+ T cells.

#### 3.2.1 CD8+ T cell-derived exosomes

The cells in the TME directly affect the occurrence, development, and metastasis of tumors through their interactions (138). CD8+ T cells play an indispensable role in the TME, and CD8+ T cells infiltrating tumor tissues are associated with the prognosis of human malignancies (139). CD8+ T cells can not only kill tumors (140, 141), but also induce the production and release of specific substances (mRNA, miRNA, protein, and lipid) by acting on recipient tumor cells, which can affect tumor development (98) (**Table 2**).

**Table 2:** Genes associated with immunotherapy in ovarian cancer.

Name	Composition	Target point (object)	Potential targets	Role	Source	Ref.
miRNA	miR-222-3p	Macrophages	Diagnostic markers	TAMs M2 polarization	Serum	(15)
	miR-92a	–	Diagnostic markers	–	Urine	
	miR-30a-5p	–	Highly specific diagnostic markers	Inhibits proliferation and metastasis of OC cells	Urine	
	miR-let-7	–	–	Inhibits cell proliferation	OC cell line	
	miR-NAs	Between the skin cells	–	Tumor cell spread	OC cells	
	miR-330–3p	Mesenchymal ovarian cancer cells	Inhibition of tumor development	Enhanced mesenchymal phenotype	Plasma cells	(142)
	miR-21	Adjacent cancer cell	Diagnosis and treatment of metastatic and recurrent ovarian cancer	Inhibits apoptosis of ovarian cancer cells	CAA/CAF	(129)
	miR-233	EOC cell	Predictors of tumor invasion, metastasis, and recurrence	Induced chemoresistance	Macrophages	(143)
	miR-6126	Tumor cells	Regulates ovarian cancer progression	Tumor suppressor factor	Malignant cells	(144)
IncRNA	UCA1	Cancer cells	Biomarker	Inhibition of cell metastasis	Urothelial carcinoma	(145)
	H19	mRNA	Markers of ovarian cancer development	Apoptosis of OC cells was induced	–	(146)
	HOTAIR	LSD 1/REST	Predictive and diagnostic biomarkers	Promotes OC cell proliferation	Plasma	(70)
	MALAT1/NEAT2	miRNA	Potential markers of ovarian cancer metastasis	Promotes cell proliferation, migration, and invasion	–	(147)
	MEG3	pcDNA	Biomarkers for the diagnosis of advanced cancer	Regulation of tumor suppressors	–	(148)
	NEAT1	Tumor cells	Prognostic markers for ovarian cancer	Prediction of patient survival	–	(149)
	XIST	Tumor cells	Early diagnosis, potential target of antitumor therapy	Promotes the proliferation and invasion of cancer cells and regulates the carcinogenesis of ovarian cancer	–	(150)
circRNA	CDR1as	miR-135b-5p	Ovarian cancer diagnosis and treatment	Tumor suppressor and promotes the expression of HIF1AN	Ovarian tissue	(151)
	circKRT7	miR-29a-3p	Evolutionary driver of malignancy in ovarian cancer	Promotes cancer cell proliferation and metastasis	–	(152)
	circPLEKHM3	miR-9/BRCA 1/KLF 4/AKT 1	Therapeutic targets, prognostic markers of ovarian cancer	Tumor suppressive effect	–	(153)
	cicrCELSR1	miR-1252	Promotes ovarian cancer	PTX drug resistance was affected and apoptosis rate was increased	–	(154)
	Hsa-circ-0078607	miR-518a-5p/Fas	Predicting adverse clinical outcomes in ovarian cancer	Inhibits ovarian cancer development	–	(155)
	circ-0061140	miR-361-5p	Ovarian cancer treatment indicators, miRNA sponge	Promotes ovarian cancer development	–	(155)
mRNA	CHAC1 mRNA	Cancer cells	Markers of increased risk of ovarian cancer recurrence	Affects ovarian cancer cell migration	–	(156)
	MUC16 mRNA	Tumor tissue	Markers of poor prognosis in ovarian cancer	Suggests an abnormal increase in MUC16	–	(157)
	MACC1 mRNA	Tumor tissue	Prognostic markers for ovarian cancer	Affects ovarian cancer migration, invasion and expression	–	(158)
	GSK3β mRNA	Tumor cells	Predicts chemotherapy sensitivity	Inhibits the development of ovarian cancer	–	(159)
	CEBPA mRNA	Cancer cell cytoplasm	Ovarian cancer diagnosis, evaluation, prognostic markers	Affects the pathogenesis of ovarian cancer	–	(160)

Endometrial cancer (UCEC) is one of the most common gynecological malignancies, with approximately 200,000 cases diagnosed worldwide annually (161). Despite the rapid development of drug therapy, the prognosis of UCEC is getting worse, and the 5-year survival rate of advanced patients is less than 30% (162). A previous study investigated the mechanism of UCEC development, and revealed the inhibitory effect of CD8+ T cell-derived exosomes on UCEC development (99). CD45RO-CD8+ T cell-derived exosomes inhibit UCEC development through the ERβ/miR-765/PLP2/Notch pathway, and these exosomes interact with the miR-765/PLP2 axis to inhibit estrogen promotion of UCEC development.

Other studies have found that miR-150 contained in CD8+ T cell-derived exosomes can act on macrophages, which in turn act on regulatory T cells. miR-150 is transferred to effector T cells to inhibit cell proliferation and the occurrence of specific immune responses (163). Some research teams have studied ovalbumin-specific TCR transgenic OT-I mice (164), and found that the exosomes derived from CD8+ T cells of mice carried ovalbumin specific TCR and FasL. These exosomes can regulate the pMHC-I expression and the FasL/Fas interaction *in vitro* by inducing DC apoptosis. We have also found that CD4+T cell-derived exosomes from ovalbumin-specific TCR OT-II mice also carried ovalbumin-specific TCR and FasL and could inhibit CD8+ CTL responses. The team demonstrated the immunomodulatory effects of CD8+ T cell and CD4+ T cell-derived exosomes using transgenic mice, but the factors responsible for inhibition have not yet been identified. We can speculate that exosomes derived from CD8+ and CD4+ T cells carrying FasL may affect immune cells through antigen-specific functions. Additional experimental data suggest that FASL-mediated apoptosis of T cells carried by exosomes is associated with tumor escape (165). Ovarian cancer-derived exosomes may impair anti-tumor immunity by carrying FasL/Fas (166), and FasL on ascites-derived exosomes in patients with epithelial ovarian cancer, as well as TRAIL, affects the presence of membranous forms of related ligand and partially explains lymphocyte apoptosis (39). Cells in the ascites of epithelial ovarian cancer lack the membranous form of FasL and are unable to make cell-to-cell contact, thus inhibiting the mechanism of Fas-induced cancer cell death. Meanwhile, exosomes promote tumor cells to attack immune cells carrying Fas by releasing complete secreted intracellular FasL, which is conducive to the immune escape of cancer cells (167).

Seo et al. found that exosomes derived from activated CD8+ T cells can regulate the cells surrounding the tumor and inhibit the development of malignant tumors (168). CD8+ T cells’ exosomes inhibit cancer development by killing the surrounding mesenchymal cells, and destroying the tumor stroma (169). Meanwhile, exosomes also act on other anticancer CD8+ T cells. Primarily, IL-12 stimulates changes in the number and size of derived exosomes by acting on CD8+ T cells, and promotes the production of granzyme B and interferon-γ by bystander CD8+ T cells (170). Li et al. found that T cell-derived exosomes can act directly on malignant tumors and exert anticancer effects. Qiu et al. showed that active T cell-derived exosome PD-1 (protein) effectively prevented T cell-mediated immune responses by binding to PD-L1 on cancer cells (171).

#### 3.2.2 CD4+ T cell-derived exosomes

CD4+ T cell-derived exosomes play a variety of roles in the TME and cellular responses. Exosomes derived from active CD4+ T cells contain various proteins (e.g., lysosomal-associated membrane protein 1, CD4, TCR) that can inhibit the antitumor immune response and cytotoxicity of CD8+ T cells, as well as inhibit the proliferation of CD4+ T cells. Indeed, exosomes derived from CD4+CD25+FOXP3+ T cells contain the anti-inflammatory mediator CD73, which inhibits the proliferation of CD4+ T cells. Exosomes containing FasL have also been derived from human B cell-derived lymphoblastoid cell lines and CD4+ T cells to induce apoptosis of target T cells (100).

Some research teams believe that CD4+ T cell-derived exosomal miRNA-150 represents the best potential biomarker for lymphocyte activation. miRNAs from exosomes derived from CD4+ T cells are significantly different from the intracellular miRNAs of other cells, and the signal of lymphocyte activation can be transmitted to serum miR-150, suggesting that miRNA-150 released from CD4+ effector T cells could be used as a serum biomarker of lymphocyte activation (101).

Exosomes secreted from CD4+ T cells carry antigenic MHC-II peptide complexes, which can act as “mini APCs” to directly or indirectly act on T cells and contribute to T cell activation (172). Regulatory T cells are known to inhibit immune cell activation, proliferation, and cytokine secretion in a non-MHC-restricted manner (e.g., DC, NK). However, CD4+CD25+ regulatory T cells can negatively regulate autoimmune responses, and exosomes derived from these cells can also exert immunosuppressive effects (173).

#### 3.2.3 CAR-T immunotherapy

At present, the research on genetically engineered T cells expressing chimeric antigen receptor (CAR) is developing rapidly. Many studies have demonstrated that allogeneic T cells or somatic cells expressing T cell receptors (TCRs) or chimeric antigen receptors (CARs) can be used for cellular immunotherapy, and are expected to become a promising therapy for the treatment of hematological and non-hematological malignancies in the future (174–176).

CAR-T cell-based cellular immunotherapy, also known as CAR-T therapy, can induce rapid and long-lasting clinical responses (177). CAR-T cell-derived exosomes are considered potential new antitumor therapies because of their high inhibitory effect on tumor growth and safety (178). The downside is that CAR-T therapy has a high potential for side effects such as acute toxicity (174).

The therapeutic mechanism of CAR-T therapy is to target cancer cells with specific T cells that are extensively cytotoxic (179). The CAR consists of a target binding domain and a transmembrane signaling domain. The target binding domain is an extracellular domain formed by CAR-T cell-specific expression, while the transmembrane domain is the intracellular domain that provides activation signals to T cells. In general, the targeting specificity of CARs is achieved through antigen recognition regions in the form of single-stranded variable fragments (scFv) or binding receptors or ligands in the extracellular domain, whereas T cell activation functions are achieved through the intracellular domain (180–182).

CAR-T therapy produces toxicity that is different from that of conventional chemotherapy, monoclonal antibody (mAb), and small-molecule targeted therapies (183). The two most common toxic effects of CAR-T immunotherapy are cytokine release syndrome (CRS), characterized by high fever, hypotension, or multiorgan toxicity, and CAR-T-associated encephalopathy syndrome (CRES), which is characterized by a toxic encephalopathy state and usually presents with symptoms such as paranoia and confusion (183, 184). Researchers have observed strong CAR T cell responses in patients with hematological malignancies, but limited CAR T cells in solid tumors. The reason may be that there are obstacles in the TME of solid tumors, such as up-regulation of inhibitory receptors (IR), which can react with homologous ligands of CAR-T cells, such as PD1 and CTLA-4, to inhibit the therapeutic response of CAR-T therapy (185). Meanwhile, exosomes derived from CAR T cells have been found to carry CAR on their surfaces (186). The exosomes carrying CAR did not express PD1, and the antitumor effect of exosomes was not impaired after recombinant PD-L1 treatment (187). CAR-derived exosomes have been shown to be safer than CAR-T therapy in preclinical *in vivo* models of cytokine release syndrome. Researchers believe that exosomes could be used to create biomimetic nanovesicles, which could be a new and effective strategy for cancer treatment (174).

Cytotoxic T lymphocyte (CTL)-derived exosomes contain CD3, CD8, and TCR, which can unidirectionally deliver lethal content to target tumor cells (188). The conjugation formed by the interaction of TCR with antigen/MHC has been found to mediate the death of target cells, and the activation of TCR promoted CTL to derive exosomes. Lethal compounds in exosomes (including granzyme, lysosomal enzymes, and perforin) activate the killing of target cells (189, 190). Some studies have demonstrated that TCR/CD3 and other complexes exist on CTL-derived exosome membranes (191).

Based on the biological characteristics of exosomes, CAR-T cell-derived exosomes play a direct role in immunotherapy. CAR-T cell-derived exosomes are functionally and structurally similar to synthetic drug vectors similar to liposomes, so CAR exosomes can be used as cancer targeting agents (176, 192). However, exosomes directly isolated from CAR-T cell culture medium may be heterogeneous and lose their targeted therapeutic effect because the antibody-derived scFv in the CAR structure determines the targeting specificity of CAR T cells (174).

### 3.3 Exosomes derived from B cells

Research on the promotion or inhibition of immune cells in the TME against the tumor has been gradually deepened. However, while the role of T cells has made some progress, the function of B cells is still unclear. Recent studies have shown that B cells play an important role in anti-tumor immunity (193), and numerous B lymphocyte populations (naive B cells, memory B cells, activated memory B cells) have been found in the TME. B cells are the second adaptive immune cell population found in TME (194, 195). B cells have been known to be carcinogenic for decades, but recent studies have linked their presence to improved prognosis in patients with cancer (196).

DC vaccines with exosomes as antigens have been shown to stimulate the clonal expansion response of T cells by pulsed diffuse B lymphocyte-derived exosomes, thereby promoting the secretion of IL-6 and TNF-α, while inhibiting the production of immunosuppressive cytokines IL-4 and IL-10 (102). However, it is puzzling that exosomes derived from B cells instead induce apoptosis of CD4+ T cells (197). Exosomes derived from heat-shocked B cells are rich in HSP60 and HSP90, and also express high levels of MHCI, MHCII, CD40, and other immunogenic molecules, and then induce antitumor effects of CD8+ T cells through these markers (125). Subsequent studies have shown that exosomes as antigens of DC vaccines have limited anti-tumor efficacy in clinical immunostimulation trails. There is increasing experimental evidence that exosomes exert immune escape effects. Mechanistically, tumor-derived exosomes may promote B lymphocyte responses (e.g., amplification of immunosuppressive B cell populations), thereby facilitating cancer cells evasion from immune surveillance (68).

### 3.4 Exosomes derived from NK cells

Clinical studies on NK cells have found that they show rapid immunity against metastatic or hematologic malignancies, as well as possessing antitumor properties (198–200). Exosomes derived from NK cells have been shown to have tumor-homing ability in a variety of tumor animal models (201), that is, exosomes can be observed in tumors within minutes to hours. Exosomes are then ingested by tumor cells inside tumor tissues, where they kill tumor cells through a variety of mechanisms. Recently, there has been a major breakthrough in the study of NK cell-derived exosomes.

NK cell-derived exosomes have two main functions (202). The first is the cytotoxic effect. The exosomes derived from NK cells contain a variety of bioactive molecules, such as cytotoxic proteins and microRNAs (203). Additionally, exosomes derived from NK cells can also be used as a carrier of anti-tumor drugs. Exosomes take advantage of the targeting of related tumors to reach tumor tissues quickly and precisely, and then increase drug concentrations. The cytotoxic proteins contained in exosomes, such as perforin and Fas/FasL, can cause apoptosis of tumor cells, but do no harm to normal cells (103, 204). Indeed, exosomes derived from NK cells are cytotoxic to melanoma cells but have no effect on normal cells (205). In this way, we can use FasL inhibitor to reduce its toxic effect on melanoma cells. Researchers have studied the principle of NK-exosomes killing melanoma cells (206), and tested the tumor-suppressive effect of NK-exosomes *in vivo* using a mouse model. It was found that the tumor size of the NK exosome-treated group was significantly smaller than that of the control group, indicating that NK cell exosomes induced the apoptosis of melanoma cells *in vitro*. The cytotoxic effect of NK-exosomes is expected to be used in the immunotherapy of cancer (207). Meanwhile, microRNAs contained in exosomes can down-regulate the expression of related genes, thereby inhibiting cell proliferation and inducing apoptosis of tumor cells (208, 209). NK exosomes also contain a variety of immune components, which can exert immunomodulatory effects by targeting the immune system through the paracrine pathway or circulatory system, and can reverse tumor immune suppression (104, 210). Basic experiments have found that NK-exosomes can stimulate immune cells (211). Additionally, NK-exosomes can reduce the immunosuppressive effect of tumor cells, which may be related to their ability to inhibit the expression of programmed death receptor (PD-1) on T cells (212).

As mentioned above, NK-exosomes are cytotoxic to tumor cells but harmless to normal cells. Indeed, in 2002, Italian scientists first discovered that NK cell-derived exosomes expressing FasL (apoptosis-related factor ligand) and perforin molecules were able to kill several types of cancer cell lines (213). However, when NK-exosomes were used against normal cells, no cytotoxicity was observed. This selective killing effect is another advantage of NK-exosomes (214, 215), as we know that traditional chemoradiotherapy methods will inevitably cause damage to normal cells while removing tumor cells. The second advantage of NK-exosomes is that they have fewer side effects. Cell therapy (including infusion) based on NK cells can cause cytokine release syndrome (CRS), referred to as a “cytokine storm,” which can trigger a variety of common factors, lead to suspension of treatment, and in some cases, may even be life-threatening (216). However, NK-exosomes have only a small chance of exploding this side effect. The third advantage is that NK-exosomes can penetrate the “protective barrier” of cancer cells. Immune cells such as NK cells cannot easily cross the “natural barriers” in human tissues, such as the blood–brain barrier, blood–testosterone barrier, and placenta, due to various factors, including the size of the cells themselves. However, cancer cells can nest in those areas and escape immune attack. NK-exosomes are nanoscale in size and contain the same cancer-killing molecules as NK cells, but they are much smaller and better able to penetrate into tumors, conferring them advantages over using cell-based therapies (178).

The characteristics and advantages of NK-exosomes have led to numerous studies on their clinical application in tumor therapy. However, researchers have struggled to isolate functional NK-exosomes on a large scale. Further study has shown that NK cells can be incubated in exosome-free medium for 48 h, before using polymer precipitation combined with density gradient centrifugation to separate EVs (217). However, this method is time extensive. Therefore, a novel microfluidic system has been proposed by the Cancer Research Center team, who found that NK cells could be captured on a graphene oxide microfluidic chip they developed. These NK cells were then incubated on the chip for a period of time, prompting them to release exosomes, which were then captured by tiny magnetic beads from ExoBeads coated with exosome-specific antibodies. The beads were removed from the chip and then NK exosomes were separated from them using a different process (218, 219). This microfluidic system holds promise for use in NK-exosome-based immunotherapy.

However, NK cells can also use tumor-derived exosomes to induce cancer cells to evade immune surveillance. Hepatocellular carcinoma cells secrete CircUHRF1 to promote the expression of mucin domain 3 (Tim-3) and T cell immunoglobulin and inhibit the secretion of IFN-γ and TNF-α by NK cells to achieve immunosuppression (66). In ovarian cancer, NK cells ingest exosomes in ascites and perform phosphatidyl-serine (PS) treatment on the surface of exosomes to internalize exosomes and induce ovarian cancer cells to evade immune surveillance (220). Additionally, there is a bidirectional effect between NK cells and exosomes in inducing immune escape. It has been demonstrated that NKG2D ligand (NKG2DL) released by exosomes in the extracellular environment mediates cancer cell immune escape using two pathways (221). NKG2D belongs to the C-type lectin-like activated receptor, which is expressed on NK cells, CD8+T cells, and some autoreactive or immunosuppressive CD4+T cells, and can detect and recognize cancer cells. MICA is the most polymorphic in NKG2DL. By expressing MICA*008, exosomes induce and activate NK cells to exhibit an immunosuppressive function, causing sustained downregulation of NKG2D after long-term stimulation, thus destroying the NKG2D mediating function. However, the release of NKG2DLs in the extracellular environment controls the cell surface expression mechanism and directly induces cancer cells to evade the immune surveillance of NKG2D. Tumor-derived exosomes utilize a T cell-independent mechanism to inhibit the killing effect of NK cells on cancer cells. Interleukin-2 (IL-2) plays an important role in the proliferation and differentiation of NK cells. Indeed, tumor-derived exosomes induce IL-2 reactivity to regulatory T cells and inhibit its access to cytotoxic cells, thus facilitating the escape of cancer cells. This dual mechanism of action reveals the role of exosomes in evading tumor immune surveillance (222).

### 3.5 Exosomes derived from dendritic cells

With the advance of research, DCs have been found to play an indispensable role in the TME. DCs are rich in alpha-fetoprotein, which can activate acquired and innate immune responses and have unique antigen presentation (absorption and expression of tumor antigens) capacity (223). They occupy a high position in tumor immunity and have been applied in the direction of cancer immunotherapy. Exosomes derived from DCs (DEX) have been found to activate the antigenic specificity of cells, induce an anti-tumor immune response, and restore the TME at the same time (224). Compared to immature DCs, mature DCs possess stronger capability in secreting exosomes that induce antigen-specific immune responses. Exosomes derived from mature DCs are 50–100 times more effective than exosomes from immature DCs when exerting immune effects *in vitro* and *in vivo* (225). DC exosomes can also be used as carriers to transmit DC antigens (226). Conversely, tumor-derived exosomes can be used as the intermediate of CTL cross-initiation (227). Exosomes take up tumor antigens and pass them to DCs to control their presentation to MHC-I molecules and induce CD8+T cells to produce effective anti-tumor effects. Meanwhile, exosomes from the ascites of metastatic patients with ovarian cancer interact with DCs to induce tumor-specific cytotoxicity and effectively kill cancer cells. Exosomes deliver tumor-specific antigens to DCs in cord blood, thus stimulating the proliferation and differentiation of resting T cells and inducing cytotoxicity to kill ovarian cancer cells, which may be a promising immunotherapy for ovarian cancer (228).

However, DCs have a poor absorption rate of tumor antigens and low immunogenicity of antigens. Under the inhibition of T cells, DC-derived exosomes are ineffective in tumor treatment (229). Moreover, DC-based immunotherapy is limited by an insufficient immune response, which makes eradication of solid tumors difficult (230). Under further study, new progress has been made, and it has been found that DC-derived exosomes are ideal antigens for DC vaccines (212).

DCs not only have antigen presentation function, but also have anticancer effects by stimulating a large number of exosomes (231). DC-derived exosomes contain MHC I, MHC II, CD86, and HSP70–90 mixtures, which activate CD4+ and CD8+ T cells (232). *In vivo*, tumor peptide-pulsed DC-derived exosomes have been shown to induce specific cytotoxicity of T cells and inhibit or eradicate mouse tumor cell growth in a T-cell-dependent manner. A vaccine regimen based on DC-derived exosomes can replace DC adoptive therapy to a certain extent (233). It is well known that the effector function of CD8+ T cells decreases (a process of depletion) upon sustained antigen stimulation, resulting in a dysfunctional immune response in the TME (105). Studies have found that DC vaccine induces anti-tumor immunity by the following mechanism: on the premise of exosomal CD80 stimulation and IL-2 secretion, the exosomal peptide MHCI begins to express, which transmits signals to CD8+ T cells to activate cell proliferation, thus inducing efficient anti-tumor immunity (2).

Additionally, exosomes derived from DCs containing alpha-fetoprotein (AFP) have been shown to induce IFN-Y-expressing CD8+ T cells in HCC mice, resulting in increased IFN-y and IL-2 and decreased CD25+Foxp3+ Tregs, IL-10, and TGF-B content (234). At present, there are different opinions on the relationship between MHC-containing DEX and T-cell responses. Most believe that DEX containing MHC activates T cell responses, while others believe that in the presence of intact antigens, DEX containing MHC is not associated with T cell responses (235). Therefore, the immune effects of exosome-based DC vaccines still need to be studied. The immunosuppressive effect of exosomes on DCs also requires attention. Czystowska et al. reported the discovery of an exosome that carries a specific substance (ARG1) and inhibits immunogenesis in the ascites and plasma of patients with ovarian cancer. Exosomes carrying special substances are transported to the draining lymph nodes and then taken up by DCs, thereby blocking their induction mechanism and ultimately inhibiting the proliferation of antigen-specific T cells and causing immunosuppression (236). Additionally, exosome-mediated IFITM2 protein (transmembrane protein 2) transport to DCs leads to inhibitory activation of the IFN-α (interferon) pathway, which reduces IFN-α synthesis and blocks the anti-HBV (hepatitis B virus) efficacy of IFN-α. As a result, the IFN pathway treated with exogenous IFN-α appears a response barrier. This study provides a new explanation for the clinical phenomenon of poor response to IFN-α treatment in CHB (chronic hepatitis B) patients (237). Moreover, a previous study showed that tumor-derived exosomes inhibited DC differentiation by acting on DCs, blocking their immune function, and showed a strong immunosuppressive effect, which may be one of the main mechanisms of immune monitoring of tumor escape (127).

### 3.6 Exosomes derived from neutrophils

Neutrophils are among the most abundant white blood cells in the immune system and are involved in forming the first line of defense in the innate immune response (238). Neutrophils play important roles in angiogenesis, immunosuppression, and cancer metastasis (239). Some research teams have suggested that neutrophils are involved in the mechanism of promoting cancer metastasis, and confirmed the feasibility of neutrophils as a potential marker of diagnosis and prognosis and a clinical therapeutic target (240). Consequently, the study of exosomal vesicles derived from neutrophils has also been put on the agenda.

Zhang et al. (106) demonstrated that exosomes derived from neutrophils (N-Ex) can induce apoptosis of tumor cells by transmitting cytotoxic proteins and activating the caspase signaling pathway. The research team developed a simple and efficient preparation method for N-Ex and NNVs, which can be used as a safe vehicle for tumor target therapy. They attempted to modify N-Ex with superparamagnetic iron oxide nanoparticles (SPIONs) and found that the modified exosomes significantly improved the efficacy of tumor target therapy. Neutrophils have also been used to produce high-yielding exosome-like nanovesicles (NNVs). Zhang et al. found that engineered SPION-NNVs can be widely and efficiently used in clinical transformation, which has great application significance in the field of drug targeted delivery and tumor therapy. As persistent inflammation is a major feature of the TME, targeted therapy of the inflammatory TME is a research hotspot (241, 242). Some researchers have developed the NEs-Exos system for glioma using N-Ex as delivery vehicles for doxorubicin (107). This treatment system does not have the limitations of conventional chemotherapy and is a promising treatment approach. Studies of N-Ex have further revealed that the activity of other immune cells, such as macrophages and T cells, can be affected by exosomes. At the same time, Li et al. found that N-Ex affected the formation of pathological blood vessels by inhibiting the proliferation and migration of endothelial cells (243). Some research teams have elucidated the potential oncogenic mechanism of exosomes in gastric cancer (244). It was found that gastric cancer cell-derived exosomes (GC-Ex) induced neutrophil activation and extended survival time. Meanwhile, the derived exosomes contain HMGB1 protein, which activates the NF-κB pathway through the interaction with toll-like receptor 4 (TLR4), promotes the autophagy of neutrophils, and ultimately induces the migration of gastric cancer cells. Other studies have shown that activation of TLR4 can stimulate the release of highly immunosuppressive exosomes, promote tumor development, and help tumor cells evade immune surveillance (245). However, the therapeutic mechanism of neutrophil-derived exosomes in ovarian cancer has not yet been elucidated.

### 3.7 Exosomes derived from macrophages

Macrophages account for approximately half of the total tumor cells (246). In the TME, the vast majority of macrophages are programmed to promote primary tumor development and metastasis (247). However, they also participate in the regulation of anti-tumor adaptive immune response and inhibit tumor growth. Ascites is an obvious indicator of ovarian cancer, which contains a large number of specific macrophages, and these tumor-associated macrophages (TAM) have certain clinical value (248). Many studies have demonstrated that TAM-derived exosomes are involved in the regulation of immune responses and cancer biology.

TAM-derived exosomes release miRNAs that act on CD4+ T cells and induce Treg/Th17 imbalance, and then directly form an immunosuppressive microenvironment to promote the development of ovarian cancer (108, 249). Studies have found that M2 macrophages secrete large amounts of exosomes with immunosuppressive activity, thereby increasing drug resistance and promoting tumor development (250). Another team found that M2-TAM-derived exosomes promote the formation of vascular mimicry in tumor cells and promote tumor development and metastasis, thereby increasing tumor aggressiveness (109). This is because miR-193a-5p carried by exosomes can specifically adsorb and down-regulate the protein expression of TIMP2 to promote the formation of vascular mimicry. Xenotransplantation models have shown that M2 macrophages-derived exosomes carrying miR-155-5p can upregulate IL-6 and affect its stability by disrupting ZC3H12B-mediated mechanisms, that may induce immune escape and tumor formation in colon cancer (251). Macrophage-derived exosomes provide miRNA delivery to ovarian cancer cells, which in turn modulates the tumor immune mechanism in ovarian cancer. These exosomes are enriched in miR-29a-3p, a member of the miR-29 family, that functions essentially during lymphocyte differentiation. High levels of miR-29a-3p expression inhibit PD-L1 expression in ovarian cancer cells by downregulating the FOXO3-AKT/GSK3β axis, leading to immune escape of OC cells and ultimately promoting the proliferation of ovarian cancer cells (252). Xu et al. conducted experiments on exosomes secreted by TAM and found that when exosomes were used as carriers to deliver antigens to DC, T cell immune responses were significantly enhanced (2). These results suggest that TAM-derived exosomes can serve as potential carriers for the exchange of cellular components between immune cells and enhance immune responses. A recent study found that exosomes secreted by TAMs contain HIF-1α stable long non-coding RNA (HISLA), which has the ability to regulate aerobic glycolysis and anti-apoptosis of cancer cells (110). This study demonstrates that RNA-interference-mediated silencing of HISLA may be a potentially powerful means to inhibit glycolytic processes in cancer cells, and they demonstrate that targeting TAMs-specific lncRNAs has great potential in cancer therapy. Nevertheless, further studies are needed to explore the interactions between TAM-derived exosomes and other immune cells and their relevance in ovarian cancer immunotherapy.

### 3.8 Exosomes derived from mast cells

Exosomes derived from mast cells (MCs) play a biological logic role in RNA and protein transfer, cell-to-cell communication, and immune regulation (125). It has been suggested that the transfer of miRNAs from MC-derived exosomes to target cells may affect intestinal barrier function (253). However, recent studies have found that lung cancer cells can absorb MC-derived exosomes, which then promote the proliferation of cancer cells by transferring KIT protein (111). The relationship between MC-derived exosomes and lung epithelial tumor cells has been explored, and morphological analysis revealed a phenotype resembling an epithelial-to-mesenchymal transition in A549 cells, which receive signals from exosomes (254). At the same time, the transcriptional analysis revealed that EMT-related phosphorylation cascades were significantly increased in epithelial cells treated with MC-exosomes (255). Other studies have found that MC exosomes can change the biological functions of DCs, T cells, and B cells. MC-exosomes induce antigen-specific immune responses by enabling T cells to produce antigen presentation capabilities (256). CD63+ and OX40L+ exosomes derived from MCs promote the proliferation and differentiation of CD4+ Th2 cells through the interaction of OX40L-OX40 (257). At present, the role of MC-exosomes in the TME is still under study, but they are expected to be a powerful means for the treatment of ovarian cancer in the future.

## 4 Exosomes as immunotherapy for ovarian cancer

Immune cells can be manipulated ex vivo to adjust the function of T cells (258), B cells, and NK cells, as well as to impart tumor destruction effects. Meanwhile, exosomes derived from stem cells also play a significant role in the field of cancer immunotherapy (259). Numerous studies have shown that stem cell-derived exosomes promote tumor growth and metastasis. Indeed, exosomes derived from mesenchymal stem cells in gastric cancer tissues promote the proliferation and metastasis of cancer cells by transferring miRNA into human gastric cancer cells, thus promoting the development of gastric cancer, suggesting that stem cell-derived exosomes can be used as a new biomarker for gastric cancer (260). The mesenchymal stem cell biomarker (MSC marker) CD105 is expressed by tumor-initiating cell subsets in renal cell carcinoma, and its derived exosomes promote cancer development. During tumor development, derived exosomes accelerate the formation of pre-metastatic niches by promoting cancer cell proliferation and migration, gene remodeling, and triggering angiogenic switches (261). Additionally, exosomes derived from internalized adipose-derived mesenchymal stem cells inhibit the proliferation of SKOV-3 and A2780 ovarian cancer cells in ovarian cancer tissues. Exosomes are involved in inhibiting the development of ovarian cancer by activating apoptosis signals and blocking the cancer cell cycle. Adipose-derived mesenchymal stem cell-derived exosomes carry miRNA to participate in cancer cell inhibition or progression, suggesting that exosomal miRNA plays an important role in the mechanism of ovarian cancer inhibition (262). The role of stem cell-derived exosomes in tumor immunotherapy is multifaceted. Although there have been studies on exosomes derived from stem cells in different tumors, studies on exosomes in the immunotherapy of ovarian cancer are limited, and they are still of great research value.

Exosomes in the serum of patients with ovarian cancer can promote the role of regulatory T cells and inhibit the effect of immune system on tumors by expressing a variety of immunosuppressive factors, such as TGF-β1 and IL-10 (263, 264). Additionally, exosomes isolated from the ascites of patients with ovarian cancer can promote apoptosis of peripheral blood lymphocytes and DCs (265, 266). Data have also shown that EpCAM and CD44 are highly expressed in ascites exosomes, serving as a theoretical and experimental basis for the application of exosomes in ovarian cancer immunotherapy (267).

As ovarian cancer is immunogenic, exosome-based immunotherapy is an attractive field of research (268, 269). In 1996, Raposo et al. first published a report on the function of exosomes in acquired immunity. Subsequently, they conducted a number of studies on the use of exosomes as non-antigenic carriers *in vivo* to stimulate T cells to produce specific immune responses so as to achieve long-term and tumor-specific immune protection (270, 271). Big data on the survival rate of patients with ovarian cancer show that the 5-year survival rate of patients with T-cell infiltration is significantly higher than that of patients without (272, 273). DC cell-derived exosomes have great application prospect in the field of ovarian cancer immunotherapy and their role in tumor antigen vaccine should not be ignored. DCs present antigens to specific T cells to activate T cell proliferation and destroy cancer cells. In addition to activating T cells, exosomes derived from mature DCs can also induce other antigen-presenting cells to activate T cells. However, exosomes derived from immature DCs have the opposite effect and increase cancer cell tolerance (274). Therefore, the DC maturation state determines whether the relevant exosomes launch immune attacks or induce tolerance. DC-derived exosomes recreate the TME while activating the cell’s antigen-specific immune response (224). Derived exosomes are also ideal antigens for DC vaccines (212). DC exosome vaccine may replace DC adoptive therapy (233), which has potential clinical application prospects. Phase I clinical trials of DC-derived exosomes have been conducted, focusing on the feasibility of exosome-presenting protein-loaded histocompatibility complexes (275–277). Researchers have hypothesized that exosomes in ascites combined with TLR3 stimulants might prolong progression-free survival in patients with high-grade ovarian cancer (278). Tumor antigen-specific T cells are naturally present in patients with ovarian cancer, and infiltrated T cells have an excellent therapeutic effect in the prognosis of advanced ovarian cancer. Combined chemotherapy/immunotherapy with TLR3 agonists using ventral water derived exosomes carrying tumor-associated antigens activates and amplifies antigen-specific T cell immunotherapy mechanisms against tumor-induced immunosuppression in advanced ovarian cancer (279).

The stability of exosomes themselves is excellent, and they exist stably in the circulation of human body fluids without causing immune rejection. Studies have shown that exosomes can also increase the stability and bioavailability of a variety of drugs, and enable efficient uptake by intestinal epithelial cells and immune cells. Indeed, the combination of exosomes with curcumin can improve the solubility, stability, and bioavailability of the drug, indicating that the success rate of ovarian cancer treatment can be improved by using exosomes as immunotherapy drugs to target cells/organs. Exosomes also have immunomodulatory biological properties. Exosomes derived from DCs can activate T cells and NK cells to enhance the killing effect on tumor cells, while those released by NK cells include FASL, perforin, and NKG2D, which can kill tumor cells *in vitro* (280). Exosomes can enhance the immune response by enhancing antigen presentation or directly activating immune cells and exerting anti-tumor immune effects. Exosomes can also induce immune tolerance, including exosomes of tumor cells carrying TRAIL, galectin9, or FASL molecules, which can induce apoptosis of CD8+T cells. Indeed, FasL expression in melanoma TEXs can induce apoptosis of T cells *in vivo* (281). Ovarian cancer TEXs inhibit T cell CD3-ξ and JAK3 signaling, thereby preventing T cell activation (282). FrarIgsmyr et al. found that FasL produced by syncytial trophoblasts was released in the form of exosomes, which induced apoptosis of effector cells expressing Fas (283). Ovarian cancer exosomes contain a variety of specific proteins, and their contents change during the development of ovarian cancer, which can be used as potential biomarkers (56, 284).

In recent years, researchers have used the relationship between exosomes and the immune system to combine traditional chemotherapy with immunotherapy to develop immunotherapy for tumor treatment (285, 286). This immunotherapy targets tumor-derived exosomes as potential antigens and uses TLR3 agonists to generate long-lasting T-cell immune effect and destroy the immune tolerance of the tumor (15, 287). Another study provided a new idea for immunotherapy of ovarian cancer, showing that exosomes derived from metastatic ovarian cancer deliver tumor-specific antigens to DCs, which then stimulate T cells to differentiate and induce cytotoxicity (228, 288).

Despite the lack of relevant data on exosome-based immunotherapy for ovarian cancer, this research direction has attracted increased attention.

## 5 Discussion

Conventional treatment of ovarian cancer can lead to drug resistance, adverse reactions, and long-term complications (2). Exosomes have great potential in the field of ovarian cancer immunotherapy as potential therapeutic markers for cancer, or as a more effective, rapid, and safe vehicle for the delivery of antitumor drugs. Exosome-based immunotherapy can activate the immune system and eliminate tumor cells (289). Exosomes have immunogenicity and molecular transfer ability and most can participate in the immune response (290). Indeed, exosomes derived from CD8+ T cells and CD4+ T cells have antigen-specific functions, which affect immune cells. The essence of the effect of exosomes in different body fluids is different. Exosomes in ascites contain miR-6780b-5p (26), those in serum contain EpCAM, Claudin4 (46, 54), and those in urine contain microRNA-30a-5p (56). In other words, a variety of exosome-based approaches can be employed to treat ovarian cancer. Exosomes are not only derived from different body fluids, but also from different cells in the TME, which have different effects on the immune response. Some cells may promote and enhance the occurrence of immune response, while others may inhibit and weaken the strength of immune response. Exosomes can form a pre-metastatic niche by acting on immune cells, and their transfer of miR-21 can change the malignant phenotype of ovarian cancer cells, which is a potential treatment for metastatic ovarian cancer (291, 292). However, exosomes derived from ovarian cancer induce T-cell arrest, which allows cancer cells to achieve immune escape (121, 293). Meanwhile, cancer cells may produce more exosomes than normal cells, and the amount of exosomes produced by different cancer cells is also different (294).

Despite the increased interest in exosome research, there remain many issues to be addressed. At present, most of the immunotherapy methods based on exosomes are in the experimental stage and lack large-scale clinical trials. Additionally, exosome isolation technology is a major difficulty. As mentioned above, the number of plasma and serum exosomes in cancer is much higher than that in healthy people, which is a promising diagnostic biomarker for ovarian cancer (53). As a whole, our conclusions are mainly based on plasma samples, while the components of plasma samples are very complex, and the effect of free proteins on exosome separation cannot be ignored (71). Differential centrifugation is the most commonly used separation method (295). However, the isolated exosomes will contain more free heterologous proteins, and the detection of the number of exosomal proteins will be limited. At the same time, the types of other proteins detected will also be reduced, resulting in inaccurate difference analysis. The feasibility of exosome separation technology is very important. Some researchers have designed molecular SEC, which can isolate exosomes with high purity (296). However, it also has certain disadvantages, which can lead to contamination by some lipoprotein impurities. Therefore, a perfect exosome separation technology is urgently needed. The activation of T cell responses by DEX containing MHC is also controversial, and the immune mechanisms of exosome-based DC vaccines require further investigation (235). Additionally, the interaction between TAM-derived exosomes and other immune cells and the relevance of immunotherapy in ovarian cancer require further study.

More importantly, exosomes have a role in immune evasion surveillance, as allies of immune escape (120). Increasing data show that exosomes play a key role in the crosstalk between cancer cells and the immune system, supporting the escape of immune surveillance by inhibiting the function of T cells and NK cells (126, 222), and the activation of monocytes, inhibiting the differentiation of DCs (127), promoting the differentiation and increase of myeloid suppressor cells (128), and inhibiting antigen-specific and non-antigen-specific antitumor responses (297). Additionally, studies have shown that DC vaccines with exosomes as antigens have limited anti-tumor efficacy in clinical immunostimulation tests (68). These findings provide new insights into the mechanisms by which cancer-derived exosomes evade immune surveillance and highlight the limitations of exosome-based cancer immunotherapy. It may be possible to effectively reduce immunosuppression by targeting tumor exosomes to expand the prospects of immunotherapy. Alternatively, exosomes can be used as potential diagnostic biomarkers to selectively eliminate cancer-derived exosomes and enhance the efficacy of immunotherapy. Before using exosomes in the clinical immunotherapy of ovarian cancer, we need to investigate the side effects of exosomes in various aspects to ensure that their effective properties are fully utilized. However, the immunotherapeutic potential of exosomes is enormous.

These studies will help to explore the application of exosomes in ovarian cancer immunotherapy, so as to accelerate their application in clinical practice.

## Author contributions

QW and XG conceived the study. XG, HC, DS, and ZX drafted the manuscript. XG and QW performed the literature search and collected the data. AT, ZX, and QW helped with the final revision of this manuscript. All authors reviewed and approved the final manuscript.
